# Boolean Models of Biological Processes Explain Cascade-Like Behavior

**DOI:** 10.1038/srep20067

**Published:** 2016-01-29

**Authors:** Hao Chen, Guanyu Wang, Rahul Simha, Chenghang Du, Chen Zeng

**Affiliations:** 1 Department of Physics, The George Washington University, Washington, DC 20052, USA; 2 Department of Chemistry, Wuhan Polytechnic University, Wuhan 430023, China; 3 Department of Biology, South University of Science and Technology of China, Shenzhen 518055, China; 4 Department of Computer Science, The George Washington University, Washington, DC 20052, USA; 5 Department of Physics, Huazhong University of Science and Technology, Wuhan 430074, China

## Abstract

Biological networks play a key role in determining biological function and therefore, an understanding of their structure and dynamics is of central interest in systems biology. In Boolean models of such networks, the status of each molecule is either “on” or “off” and along with the molecules interact with each other, their individual status changes from “on” to “off” or vice-versa and the system of molecules in the network collectively go through a sequence of changes in state. This sequence of changes is termed a *biological process*. In this paper, we examine the common perception that events in biomolecular networks occur sequentially, in a *cascade*-like manner, and ask whether this is likely to be an inherent property. In further investigations of the budding and fission yeast cell-cycle, we identify two generic dynamical rules. A Boolean system that complies with these rules will automatically have a certain robustness. By considering the biological requirements in robustness and designability, we show that those Boolean dynamical systems, compared to an arbitrary dynamical system, statistically present the characteristics of cascadeness and sequentiality, as observed in the budding and fission yeast cell- cycle. These results suggest that cascade-like behavior might be an intrinsic property of biological processes.

Biologists often view cellular processes as a chain of events, especially within small subsystems in the cell. For instance, an external signal turns on gene A, which then turns on gene B, which turns on gene C, and so on. This view of molecular events is inherently *cascade-like*, suggesting a chain of causality occurring over intervals of time, where each particular change involves only a few of the molecules in the subsystem. In this paper, we will investigate this phenomenon and ask the following question: is there any inherent reason for biological processes to be cascade-like?

To answer this question, we model biological system with Boolean methods. In the last decade, Boolean models have been applied widely in systems biology^1^,^2^. In these models, time is discrete and the whole dynamical process is divided into several time steps, then the status of a particular molecule *i* at any given time step is either “on” (active, or in high concentration) or “off” (inactive, or in low concentration), and in the next time step the status of *i* is determined by the statuses of the molecules interacting with *i* in the present time step. This method was first introduced by Kauffman^3^. After that, Bornholdt and other researchers published significant work on random Boolean threshold networks^4^,^5^,^6^,^7^. Differing from the random Boolean networks used by Kauffman, the concept of activation, inhibition and self-degradation introduced in the threshold model give the model more biological significance. Indeed, several biological systems have already been modeled by the such threshold networks and those models appear to explain biological network phenomena reasonably well^8^,^9^,^10^.

As an example, let us consider the table in Fig. 1A. It represents a system of 11 molecules involved in regulating the budding yeast cell-cycle^8^. At the start, only molecules Cln3, Cdh1, and Sic1 are active; at each time step the status of individual molecule is changed under dominant inhibition rule (which is one of the threshold rules, details of which are shown in the “Methods” section) and finally, after 10 steps the system settles into a “steady state” or *attractor*. We use the term “Boolean process” to denote the sequence of these booleanized states. The sequence can be conveniently represented as a binary matrix, as shown in the table. In the following, we may use the term “process” or “binary matrix” to substitute for “Boolean process” when not ambiguous. Based on the literature of known reactions between 11 molecules, a hand-crafted^8^ biological network that determines the causality of events is shown in Fig. 1B. As it turns out, this is not the only network that explains the process—the network shown in Fig. 1C is an example; it is a minimal (fewest possible edges) network that suffices. For a given Boolean process, the number of networks that can explain the process is termed its *designability*. As a design principle, designability was first observed in the field of the protein folding^11^. And there is also evidence that biological processes have high designability^12^.

Next, looking at the diagonal in the table in Fig. 1A, one can see that most of the “1” elements in the table are clustered along the diagonal, except for molecules Cdh1 and Sic1 (which are active in off-diagonal entries). This is a typical example of the striking cascade-like sequence of events. In some ways, a “mostly diagonal” table is an approximate representation of sequentiality: the molecules towards the top left are active at the beginning of the process, and turn on others, which activate others and so on. We use the term *cascade* to refer to a Boolean process whose activity is concentrated approximately along the diagonal. Thus, a quantitative definition is proposed to quantify the “cascadeness” (how close to a cascade is a Boolean process?). Now our main question can be formulated as: is it true that most biological processes are like the cell-cycle examples, possessing high cascadeness? And if so, what is the reason? The main contribution of this paper is some evidence that design principles such as designability^12^ and robustness (defined below) naturally lead to cascade-like processes.

We now examine the two design principles in more detail:

- **Robustness**. Robustness is a widely desired property in biological systems. One way to quantify robustness is to assess the stability of a biological system to perturbations. In this paper, we consider whether a biological process is robust to perturbations, and how robustness requirements shape a process. Observing the budding and fission yeast cell-cycle, we find that: beyond dominant inhibition rule, both of them obey two additional dynamical rules which are robustness-related:

– **Reliable transition requirement**. Consider a molecule *i* that goes from “off” (inactive) in step *t* to “on” (active) in step 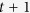 because some other molecule *j*, which was active at *t*, stimulated this state transition for *i*. It is also possible that another activated molecule *k* at *t* will inhibit molecule *j*. Now let’s see what things will happen if the above case occurs. In our Boolean model, since all molecules update their statuses simultaneously, in this case, though the status of molecule *j* will be “off” in step 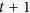, the status of molecule *i* is still “on” in step 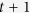 according to Eq. (1). But, in practice, a real biological system is continuous, with non-integer concentrations of biomolecules, and therefore Eq. (1) is only a simplified discrete description of it. Thus, if the inhibition of *k* to *j* is very fast, *j* does not have enough time to activate *i*. To avoid this type of instability, molecule *j* should remain active for enough time. In other words, to make a transition reliable, a stable activation/inhibition is needed, otherwise, the transition is “brittle” and the corresponding system is not robust and will likely be eliminated in evolution.

– **Convergence requirement**. The second type of robustness results from considering the trajectory of returning to the steady state after a small perturbation. It is desirable that the perturbed trajectory of states remain “close” to the original biological process, as confirmed by the previous research on trajectory perturbation for particular biological networks^8^,^13^. A very different trajectory, even if it returns to the same attractor, would suggest that the original process had no function other than the attractor; this is certainly not the case for carefully staged events in processes like the cell-cycle, where a drastically different sequence of events would not result in the normal cycle. To apply this robustness requirement, we define a new term: middle state “
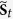
”, which represents all states between **S**_*t*_ and **S**_*t*+1_ (see precise definition in “Methods”). This definition naturally leads to the rule of “middle state to middle state” (see details in “Methods”) and fortunately, two cell-cycle systems indeed comply with this added rule.

Both of these high-level characterizations of robustness are given precise mathematical definition in the “Methods” section.

- **Designability**. As proposed in^12^, biological processes are likely to be highly designable. The reason maybe is that the processes with high designability can allow for exploration through mutations and this property is desirable for biological systems. In this paper, for a feasible process (the processes with non-zero designability), we extend the notion of designability in two ways: (1) its *minimality m* is defined to be the number of interactions in the minimal network—a network that can realize that process with the smallest number of interactions (edges). These minimal networks have been noticed in the literature^14^,^15^,^16^,^17^. In our opinion, a process with smaller *m* indicates that the core functionality of the process can be achieved with a few interactions, suggesting that process can be efficiently designed. (2) Next, we can trace the contribution to designability from each node (molecule). By identifying which one contributes the least, the minimal contribution is termed as the minimal individual designability 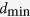. In fact, in our Boolean model, the designability *D* is a product of individual molecular designabilities 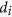, so the smallest 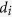 is exactly 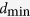. The idea is to examine whether biological networks tend to have molecules that contribute little to designability, and might therefore have been discarded by evolution.

Once we are able to mathematically characterize and computationally evaluate robustness and designability, it becomes possible to answer the questions initially raised. Our results show that the cell-cycle processes are robust and highly designable indeed. Also, if we require a Boolean process is robust and highly designable, then statistically, it must be a cascade-like process and the corresponding network is sequential, providing theoretically validity to the common perception. In the remainder of the paper, we describe the particular quantitative results of these conclusions, as well as the mathematical and methodological details (an extension of the Boolean model and technique described in^13^).

## Results

### Feasible processes are rare

First, let us consider the feasibility of a process. We ask the question: how likely is it that an arbitrary Boolean process has a network solution? We examined this question for both the case with robustness constraints (Eqs (2, 3, 4, 5, 6, 7)) and without (only Eq. (1)). Several billion such processes are generated and for each of them we checked the feasibility. Results indicated that feasibility was very sensitive to the mean activity value *a*, which is the ratio of “1” in the binary matrix (see Fig. 2). Another observation is that there is a narrow range of *a* for which feasible processes are likely, similar to the phase-transition result for the Boolean satisfiability problem^18^. For Boolean processes with 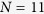 and 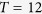 and ending with an attractor, take the case 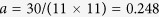 as an example (see section “Methods” for precise definition of *a*). We generated ten million processes with 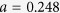 and the process with a node which has never been activated or inhibited is excluded (this criterion is also used in the following sampling or enumeration). For each process, we tested whether or not it satisfies Eq. (1). The feasibility *f* for 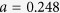 is then calculated as the fraction of processes that have solutions, which has the value 0.0141. We also examined the case 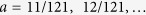 in the same manner (The processes with mean activity *a* < 11/121 are unfeasible). The results are presented in Fig. 2A. The same studies were performed for the case with robustness constraints (Eqs (2, 3, 4, 5, 6, 7)), with the results presented in Fig. 2B. Moreover, we can estimate the overall feasibility *f*_*OA*_, the ratio of the total amount of feasible Boolean processes to the total amount of arbitrary Boolean processes. In the original model, *f*_*OA*_ = 2.95 × 10^−7^; in the new model, *f*_*OA*_ = 1.72 × 10^−14^. Both values are very small and indicate that feasible processes are *rare* in the process space.

### The cell-cycle processes are highly designable

An organism undergoing evolution undergoes frequent changes at the evolutionary time scale, with adaptations that involve forming new biological functions (processes). The formation of new functions entails rewiring networks of molecular interactions. However, if a process is fragile so that any small change to the network results in dysfunction (not carrying out the original process), it is not likely to survive. Instead, the trick is to maintain the process (function) while simultaneously making small evolutionary (wiring) changes for adaptation. A process with high designability makes it more likely that a small change will merely result in another network solution that satisfies the process.

Therefore, one wonders whether known biological processes (budding yeast and fission yeast, in our examples) have a large designability as compared with other feasible processes with the same size. Here the results of budding yeast are presented, while those of fission yeast are included in Supplementary information. We randomly sampled one million processes with 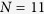 and 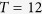 (the size of budding yeast cell-cycle process) in the space of all feasible processes and computed designability of each process; this is shown in Fig. 3A with the designability plotted against activity, where the size of each dot corresponds to the number of processes for that combination of activity and designability. One sees that the budding yeast cell-cycle process (the red triangle) has the largest designability among all the processes with 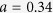 that we generated.

One can also analyze the minimal individual designability 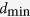. We produce Fig. 3B, which is the same as Fig. 3A except that the *y*-axis now represents 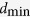. One sees that the budding yeast cell-cycle process has higher 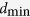 than almost all the sampled processes with 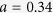. Similar results were obtained for the fission yeast (see Supplementary Fig. 2).

### The cell-cycle processes can be high-efficiency designed

Beyond designability, it is of interest to ask whether a process can be realized with an efficient network, that is, a network with few edges. In comparing two possible networks for the same process, one might reason that the network with more edges is harder for evolution to design. To see whether the budding yeast cell-cycle process is efficient in this sense, we compared it with one million randomly generated feasible processes of the same size 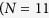 and 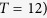. For every process, we computed the number of edges in the minimal network needed for that process (the minimality *m*). As Fig. 4 shows, the budding yeast cell-cycle process (the red triangle) has much lower minimality (with 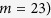 among all sampled processes. A similar result was obtained for the fission yeast model (see Supplementary Fig. 3), indicating that these two biological processes can be designed with high-efficiency.

### Robustness constraints make better processes

Recall that we imposed two robustness criteria and thus, one might ask whether there is a cost to robustness, for example, whether robust processes (here “robust process” means that a process is feasible under Eqs (2, 3, 4, 5, 6, 7), the same below) are less designable or need more edges (have higher minimality). For Fig. 5A–C we computed the designabilty *D* and minimality *m* for randomly generated processes that are robust (blue curve), and compared these metrics for random processes that are just merely feasible (red curve). Interestingly, robust processes tend to have higher designability and lower minimality.

Next, we ask the question: are the two cell-cycle processes robust? That is, do they satisfy every robustness equation among Eqs (2, 3, 4, 5, 6, 7)? To address this question, we examined each molecule in each step of these processes, and computed the fraction (percentage) of matrix entries that satisfy the conditions. The percentage of molecular states that satisfy these criteria is 99%: only one molecule in one step of the budding yeast does not satisfy the second robustness criterion (the first molecule is a trigger molecule and we do not check it). A similar result is obtained in the fission yeast and also only one molecule in one step does not satisfy the second robustness criterion. That means that both two cell-cycle processes are robust and might indicate that these two robustness criteria might occur widely in biological systems.

### Biological processes are cascade-like and sequentially activated

We now turn to the question at the beginning about whether biological processes are likely to be cascade-like. First, for any cascade, we compute the cascadeness distance *c* that measures how different a process is from a cascade (see section “Methods” for a precise definition). This cascadeness distance is shown along with designability and minimality in Fig. 5A–C. Thus, robust processes have a better (lower) cascadeness distance. Next, for further investigation, we enumerated all processes of size 8 × 9, and identified all the 112,877,531 robust processes among them. For each robust process, we calculated minimality *m*, designability *D*, and minimal individual designability 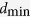. Inspiring by the earlier results on designability and minimality, we identified 89,733 processes in the top 1% with regard to *m*, *D* and 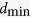 as the candidates for the biological systems. After rearranging the columns using a smart-exhaustive algorithm (see details in Supplementary information), we found that all of them are cascade-like (see the distribution of the cascadeness distance *c* in Supplementary Fig. 4). Furthermore, we clustered these 89,733 processes using the k-means clustering algorithm with 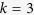. For each cluster, an “average” process is calculated and shown in Fig. 6A–C, respectively, which clearly demonstrate their cascadeness.

**Figure 1:**
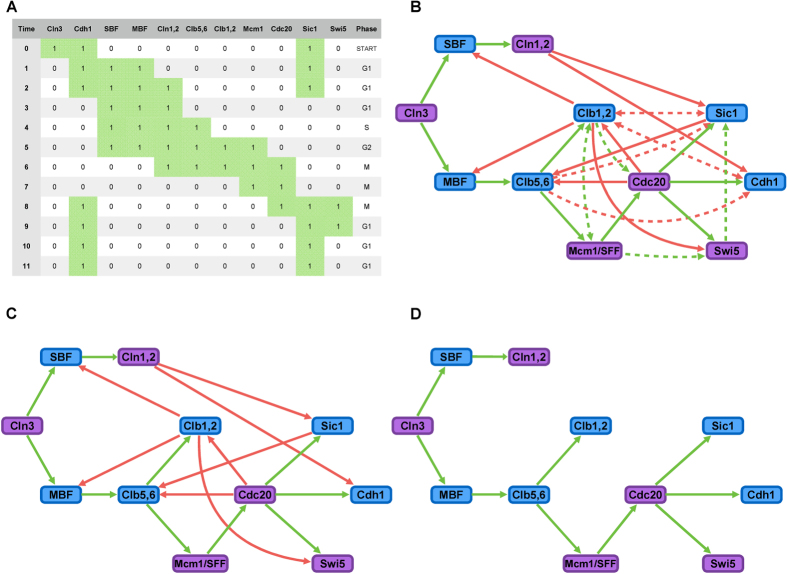
A simplified description of the budding yeast cell-cycle. The process part of the cell-cycle is shown in (**A**) and the network parts are shown in (**B**–**D**). In the process part, “0” represents that the node’s status is inactive, “1” represents active and the right column indicates the phases of the cell-cycle. In the network part, activations are shown in green arrows, inhibitions are shown in red arrows and the nodes with self-degradation are colored by purple. Starting at state of “START” phase which is shown in (**A**) and following the dynamical rule which is describe in Eq. (1), the simplified biological network which is shown in (**B**) undergoes the biological process, which is shown in the table of (**A**). Network (**B**) is not the only network, particularly, after moving some edges (shown in the dashed arrows in **B**), the remaining network shown in (**C**) can also undergoes the same process; its edges cannot be reduced. We term this network (**C**) as the backbone motif of the network (**B**), a minimal network^13^. (**A**) The time course of the 11 nodes as a representation of the cell-cycle process. (**B**) The full cell-cycle network. (**C**) The backbone sub-network contained in the full network. (**D**) Stimulation edges only of the backbone sub-network.

**Figure 2:**
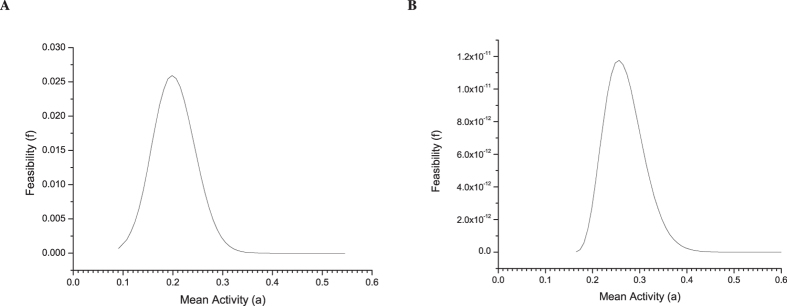
Feasibility versus mean activity. The sampled processes are all with the size of 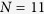 and 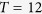 and end with a steady state. And, the process with a node which has never been activated or inhibited is excluded when sampling (the same criterion is used in the following sampling or enumeration). (**A**) The original model (satisfiability of Eq. (1)). Sampling starts with 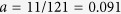 and the reason is that each node should contribute at least 1 in activity (a node with all-zero state is excluded in the enumeration approach). (**B**) The new model (satisfiability of Eqs (2, 3, 4, 5, 6, 7)). Sampling starts with 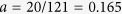 because all processes with less activity are unfeasible under the new model, which we have confirmed by enumeration.

**Figure 3:**
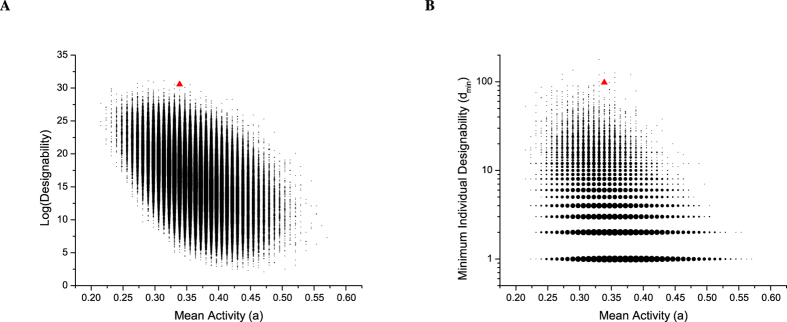
Designability versus mean activity of Boolean processes with *N* = 11 and *T* = 12. The size of each dot corresponds to the number of processes for that combination of mean activity and designability. The red triangle represents the Budding yeast cell-cycle process. (**A**) The designability in terms of Eq. (1). (**B**) The minimal individual designability in terms of Eq. (1).

**Figure 4:**
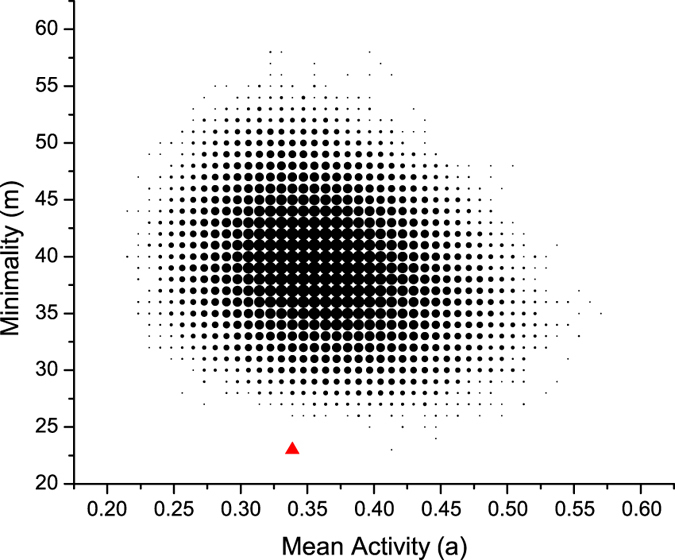
Minimality versus mean activity of Boolean processes with *N* = 11 and *T* = 12. The size of each dot corresponds to the number of processes for that combination of mean activity and minimality. Minimality is calculated under the Eq. (1). The red triangle represents the budding yeast cell-cycle process.

**Figure 5:**
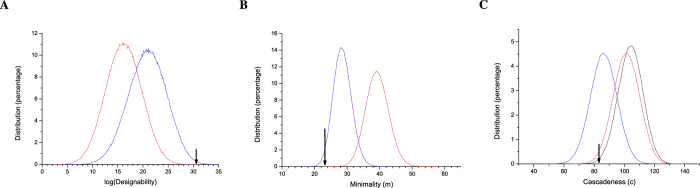
Comparison of the phenotypical features of feasible processes with and without robustness constraints. The sampled processes are all with 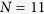 and 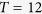. For (**A**,**B**), 1 million feasible processes are randomly generated with and without robustness constraints (Eqs (2, 3, 4, 5, 6, 7)). For (**C**), since cascadeness distance *c* is related to the activity *A*, we fix the activity 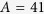. One million processes are randomly generated for each curve respectively. Red curves represent the feasible processes without robustness constraints (only satisfiability of Eq. (1)), blue curves represent the feasible processes with robustness constraints (satisfiability of Eqs (2, 3, 4, 5, 6, 7), black curve in (**C**) represents the entire random processes. The black arrow shows the position of the budding yeast cell-cycle process and it clearly shows that that the biological process has higher designablity *D*, lower minimality *m* and lower cascadeness distance *c*. To make data comparable, when calculating designability and minimality, we always use Eq. (1).

**Figure 6:**
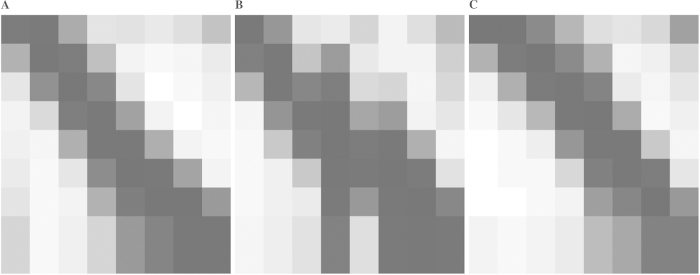
Three averaged processes of size 8 × 9. All 112,877,531 feasible processes (satisfiability of Eqs (2, 3, 4, 5, 6, 7)) are obtained via enumeration. Filtering by minimality *m*, designability *D* and minimal individual designability 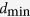, 89,733 processes are remaining. They are classified into three clusters by k-means clustering algorithm and the averaged process of each cluster is shown in (**A**–**C**) (drawn in grayscale, black means “1” or active and white means “0” or inactive).

One can also assess sequentiality by examining networks. Some networks have a layer like structure in which some molecules activate others, which activate yet others, and so on, in a feedforward fashion. Figure 1D shows how the budding cell-cycle network can be redrawn to emphasize this tree-like structure. We examined all minimal networks of each of the 89,733 processes. Among them, we find that there are 47,078 processes all of whose minimal networks have this layer-like structure, and an additional 20,399 processes have more than half of their minimal networks with this type of structure. Because this type of network structure inherently implies sequentiality, this result explains why biological processes have cascade-like behavior.

Are biological processes cascade-like? For the naturally occurring biological processes, we examined the budding yeast cell-cycle process^8^ and the fission yeast cell-cycle process^9^. After canonical re-arrangement of columns, they are both found to be cascade-like (see Fig. 1A and Supplementary Fig. 1A).

### Cascade-like processes are not necessarily biological

**Figure 7:**
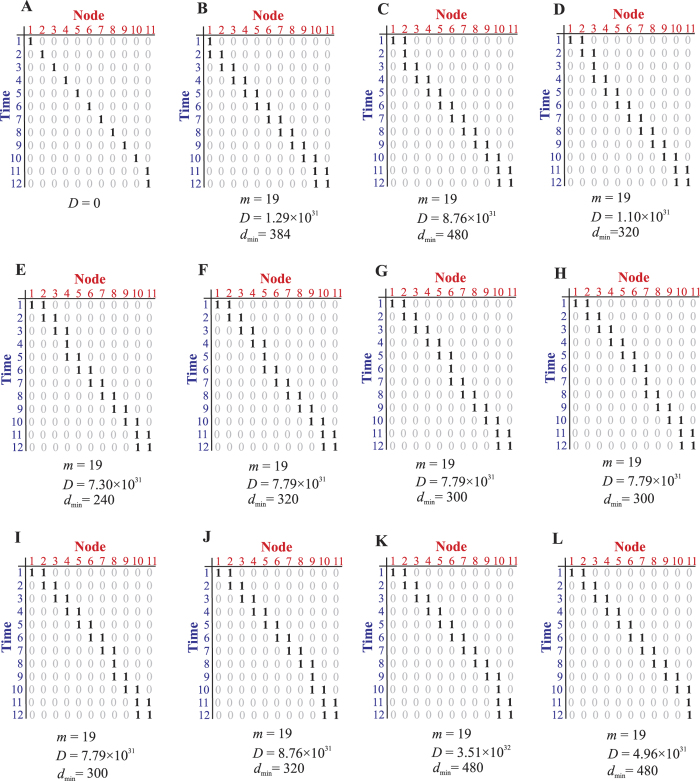
Some simple cascade-like processes. These processes all have 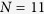 nodes and 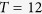 time steps. The time step 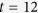 is a repetition of 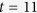, indicating the final state is a steady state. (**A**) A process with only one active molecule at every time step. The process is infeasible under Eqs (2, 3, 4, 5, 6, 7). (**B**–**L**) The simplest cascade-like processes that are feasible and their minimality *m*, designability *D*, and minimal individual designability 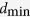 under Eqs (2, 3, 4, 5, 6, 7).

We have shown that biologically robust processes must be, in a statistical sense, cascade-like. The reverse question is also relevant: is an arbitrary cascade-like process always biological, that is, does a cascade-like process automatically satisfy all requirements from a biological system? We examine processes that are very cascade-like, including the *strict cascade*, which is when we get a simple diagonal of 1’s in the matrix (Fig. 7A). We use the set of cascade-like processes with 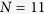 and 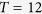 as an example to characterize cascade-like processes. For every process, the network states at 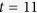 and 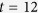 are the same, implying a steady state has been reached in the end.

Actually the strict cascade turns out to be infeasible under our robustness criteria. To ensure feasibility and avoid the process ending with a all-zero state, the minimal activity *A* is equal to 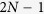 and totally there are *N* difference processes (Fig. 7B–L). Certainly, all of them are cascade-like. We call these processes the *pure cascades*. We determined their minimality *m*, designability *D*, and minimal individual designability 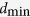 with our two robustness constraints and the results are shown in Fig. 7B–L. By these measures, the pure cascades appear to be similar to the cell-cycle processes. Unfortunately, a closer examination reveals that the dynamic properties of the corresponding networks are not suitable, as we now explain. For example, consider the last pure cascade (Fig. 7L) as an example. We studied the dynamics of each of the 1,024 minimal networks for this process (since it is impossible to simulate every one of the 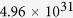 solutions for this process). The basin size *B* (range from 1 to 
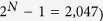
 of each network was calculated: among 1,024 networks, 384 networks with 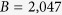, 192 networks with 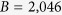, 192 networks with 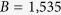 and only 256 networks with *B* < 200. At first glance, this appears to be a positive result, suggesting that the pure cascades have sufficiently large basin sizes for their main attractor state. However, these networks exhibit poor dynamics when one examines the flow of states through the process, as described in^8^. We use the term flux for a state to indicate how many paths from other states pass through the given state. A state with small flux indicates that it is only a small branch of the tree. If the flux of all nodes is small (which is corresponding to a low average flux), it means that lots of states have independent paths to the attractor, an indication of a “brittle” process. Instead, what is desired is high flux for the states in the process so that most perturbations will return back to the process^8^. For the pure cascade shown in Fig. 7L, consider the flux of the 9th time step, among all 1,024 networks. The maximum flux is only 107, indicating that most of states go through different trajectories. We also tested the other pure cascades and obtained similar results, suggesting that at least these pure cascades are not suitable as biological processes. When combined with the observations that biological processes are cascade-like, this might mean that biological processes are concentrated at the “edge” of cascadeness, where the few additional non-pure cascade “1” elements in the process confer desired dynamical properties.

## Discussion

In this paper, we investigated the common perception that events in biomolecular networks occur in a cascade-like manner. For example, both budding and fission yeast cell-cycle show the cascadeness in process-view (see Fig. 1A and Supplementary Fig. 1A) and the sequentiality in network-view (see Fig. 1D and Supplementary Fig. 1D). To analyze the phenomenon, we used Boolean methods to model biological systems. When Boolean models are augmented to include designability and robustness to meet the biological requirements, our results show that the qualified Boolean processes are statistically very likely to be cascade-like, providing a theoretical explanation to this common phenomenon.

Now, let us examine our two additional dynamical rules again: the first rule is to make every transition of the node’s status reliable and the second rule is to make the dynamics trajectory tree convergent. The two rules might seem arbitrary, but in this section we provide some arguments for their generality. First, note that though the two rules have different meanings, they have similar mathematical form (see Eqs (2, 3, 4) and Eqs (5, 6, 7)) and both of them improve the beginning model which is described in Eq. (1) via adding the additional control of the middle state “
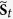
” (see definition in the section “Methods”). If a small perturbation occurs on **S**_*t*_ and leading to a middle state “
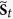
”, these rules can guarantee that this perturbation will not diverge and can converge to the same steady state. In particular:

1. The first rule requires the following: if 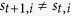, then for each 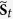, in the next step 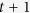, the status of *i* must be equal to 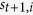. This requirement also implies that the quick flips of the node, such as “
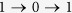
” or “
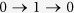
”, are forbidden. The reason is that: **S**_*t*+1_ can be also seen as a special middle state 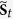, so if 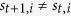, as we describe above, **S**_*t*+1_ as a member of 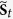, in the next step *t* + 1, the status of *i* must be equal to 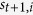.
2. The second rule requires the following: if 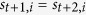, that is 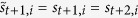, then for each 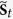, in the next step 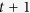, it must go to 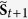, it also means that the status of *i* must be equal to 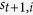.

These interpretations show that both rules are not limited to the Boolean model under dominant inhibition rule and can be easily extend to an arbitrary Boolean model. We tested them with the majority rule model of the budding yeast cell-cycle process^8^ and obtained a similar result: only one molecule in one step does not satisfy the second robustness criterion. Majority rule is another threshold rule and the difference between the models under dominant inhibition rule and majority rule is: in the former, the inhibition is dominant; but in the latter, the importance of the inhibition and the activation is equivalent. These observations indicate that both rules might be not specfic model dependent and they are applicable universally in biological systems.

Furthermore, let us turn our attention back to the middle state 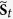. If we treat 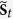 as the product of an asynchronous update, obviously, our two rules enhance the ability of robustness to this timing variations. As it turns out, this general notion has been considered by others in different contexts. For example, Peixoto and Drossel proposed the concept of “reliable dynamics”: a fully reliable process is the process that at each time step only one node’s status is changed, so it is independent of the order in which the nodes are updated^19^. It is easy to see that fully reliable process is a special case in consistent with our second robustness criterion. Mangla *et al.* proposed a similar concept, entitled “timing robustness”, to describe the ability of a system to maintain its function(process) in the presence of timing perturbations. Next, they found that both budding and fission yeast cell-cycles were highly timing-robust and the reason was due to evolutionary pressue^20^. Our work give a comprehensive understanding of why two cell-cycles systems are highly timing-robust and may have implications for the design of the system with timing robustness.

## Methods

### A Boolean model for biological processes

Boolean networks were first proposed by^3^, and then used as a theoretical model for studying inherent properties of gene regulation networks^19^,^20^,^21^,^22^,^23^,^24^,^25^. Variants of the original random Boolean network have been widely adopted by recent works^9^,^26^,^27^,^28^ to capture biological phenomena, especially for those large-scaled systems^29^,^30^,^31^ not handled by traditional methods such as ordinary differential equations.

The Boolean model in this paper is based on our previous work^13^,^32^. In the model, molecules update their statuses simultaneously in a deterministic manner and can be described succinctly as the following Boolean equation (Attn: it’s not an algebraic equation):


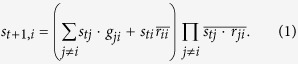


Here 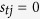 or 1 represents the status of node *j* (molecule *j*) at time *t*; it is the entry in row *t* and column *j* in the Boolean process (in the matrix or tabular form); 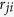 is a Boolean variable that represents a putative inhibitory (red) edge from node *j* to node *i*; similarly, *g*_*ji*_ represents a putative stimulatory (green) edge from node *j* to node *i*; addition represents the Boolean operator OR; multiplication represents AND; the bar on a variable represents NOT. The model reflects the basic rules of biomolecular interaction: an active molecule can stimulate (via a green edge) another molecule into being active, but only if no other active molecule has a red edge to the target molecule. Further descriptions are included in Supplementary information and more details are given in^13^,^32^.

To study the impact of robustness, we need to add to the model the two robustness criteria described earlier:

• **Reliable transition requirement**. First note that the activation of node *i* at time *t* (the 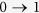 transition) depends on an activation edge from some node *j* 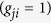 and the condition that node *j* is active at time *t* 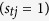. This explains the appearance of 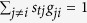 in Eq. (1). On the other hand, the 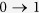 transition can be blocked by any inhibition, which explains the term 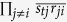 in Eq. (1). However, although 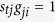 for some *j* can make the 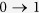 transition occur, the transition can be halted if 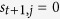. Therefore, it is desirable to have a persistently active node to enforce the activation. This stable activation can be represented as 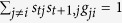. Similarly, 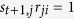 for some *j* will try to revert this transition. That means when designing a robust 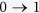 transition: we not only allow 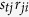 to perform the inhibition, but also 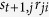. That is, 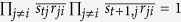 captures the above requirement of inhibition for the 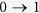 transition. By combining these requirements, one obtains Eq. (2). Similar considerations for the transition 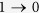 lead to Eq. (3). For the cases 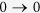 and 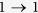, the corresponding equations (Eq. (4)) remain the same as the original ones. In summary, one has the following modified model:


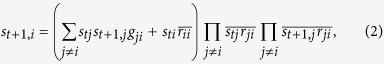


for 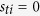 and 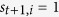



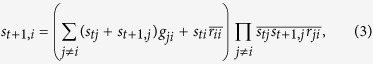


for 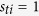 and 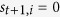



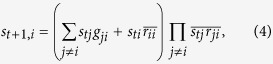


for 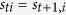.

**Figure 8:**
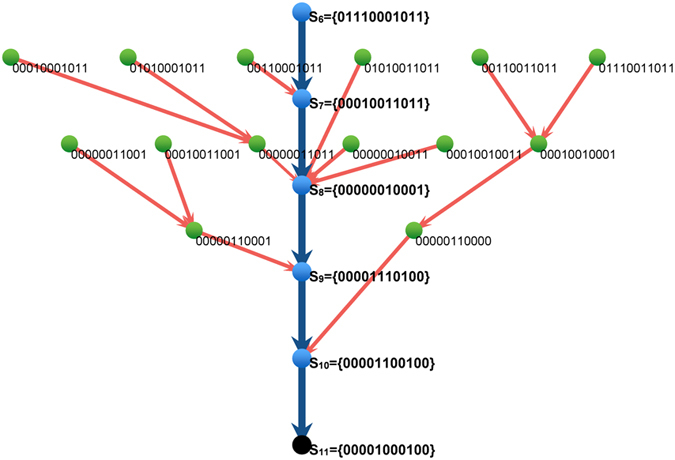
Demonstration of convergence requirement. Here we show a real case in the budding yeast cell-cycle. The black dot is the biological steady state **S**_11_, the blue dots are the states in the biological trajectory (from **S**_6_ to **S**_10_), and the green dots are the “middle states” 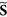. All middle states of 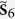 and 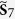 and only some of 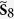 are shown in the picture. In more detail, 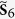 includes **S**_6_, **S**_7_ and all six green dots in the first layer, similarly, 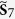 includes **S**_7_, **S**_8_ and all six green dots in the second layer. It clearly indicates that: for each 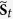, in the next time step, it goes to 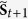 and finally it converges to the steady state.

• **Convergence requirement**. A biological process is a trajectory of state transitions. For robustness, the model ought to allow some perturbations of individual states while maintaining the trajectory. As we discuss before in the “Introduction” section, let us consider the middle state 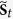 between the state **S**_*t*_ and the state **S**_*t*+1_. Here let’s give a precise definition of 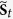. For molecule *i*, all possible statuses of *i* in 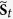 is termed as 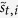 and we require that it should satisfy the following properties: if 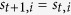 for any 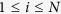, then 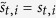; if 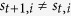, then 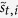 can be arbitrary. For example, if 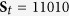 and 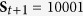, then 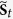 = 1 * 0 * *, where * represents arbitrary 0 or 1. Thus, there are 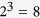 middle states 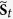, which include **S**_*t*_ and **S**_*t*+1_. For the convergence purpose, we expect 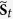 goes to 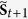 in the next step and this expectation really occurs in the budding yeast cell-cycle (see Fig. 8). If 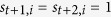, then one must have 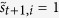. That is, no matter what 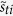 is, it must transit to 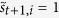. To guarantee this 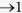 transition, one must have Eq. (5). If 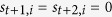, then one must have 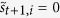. That is, no matter what 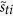 is, it must transit to 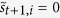. To guarantee this 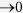 transition, one must have Eq. (6). For the cases 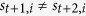, the corresponding equations (Eq. (7)) remain the same as the original ones. In summary, one has the following modified model:


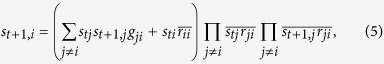


for 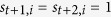



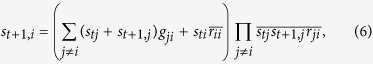


for 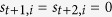



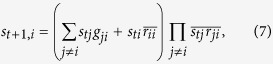


for 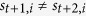.

We point out that sometimes, as is the case with Cln3 for the budding yeast cell-cycle, a molecule’s purpose is to trigger a process, in which case it appears just once in the first row and never again; in this case, we do not include it in the above analysis because it is not part of the core network.

Taken together, the above additions to the model impose some robustness: any solution of these equations results in a network that can tolerate perturbation errors as defined above. And, more importantly, for a given process, since all status variables 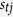 are known, it’s easy to see that Eqs (2, 3, 4, 5, 6, 7) have the same mathematical function form as the Eq. (1), that means that we don’t need to pay any additional computational cost to acquire these two robustness criteria. We can use the same analytical algorithm of the original model directly^13^.

### Phenotypical features of Boolean processes

In this paper, we are interested in examining the space of Boolean processes and asking questions about what types of processes are biological-like, and what the characteristics of biological processes are (regardless of what network actually generates the process). To this extent, we define the following characteristics or phenotypes of a Boolean process:

- **Feasibility** *f*. A feasible process refers to a Boolean process with non-zero designability. In other words, for a feasible process, we can find at least one network that can explain it under Eq. (1) or Eqs (2–7). As we mention before, we term the process which is feasible under Eqs (2–7) as robust process. Moreover, we can also define the feasibility for a given set of many Boolean processes, which is the percentage of feasible processes.
- **Activity** ***A*****/Mean Activity** *a*. These are a coarse indication of the level of activity in a process. The term activity refers to the number of molecule activations (the number of “1” in the matrix) in the Boolean process and mean activity is the percentage with respect to the size of the matrix. Notably, in this paper, we only focus on the process which is ended with an attractor, so the last repeated row is excluded from the calculation. For example, in the budding yeast cell-cycle process (see Fig. 1A), *A* = 41 and 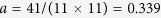.
- **Designability** ***D*****/Minimal Individual Designability** *d*_min_. The term designability is, as mentioned earlier, the number of networks that can realize a given Boolean process. Our prior work^13^ explains how this number can be computed efficiently. Note that *D* can be written as 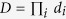, the product of the individual node designabilities^12^,^13^, it is possible that *D* is large but 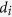 is very small for some *i*. Then, small 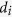 would be a bottleneck that limits mutation stability. To reflect this case, we also calculated the minimal individual designability 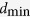, that is the smallest 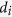, for *i* = 1, 2, …, *N*.
- **Minimality** *m*. A feasible process can be often realized by many networks. Some networks have more edges (corresponding to molecular interactions) and some have fewer. Minimal networks refer to the networks with the smallest number of edges. The number of edges in a minimal network is called the minimality of the process.
- **Cascadeness distance** *c* is used to quantify “cascadeness”, the degree to which a Boolean process is cascade-like. It is defined to be

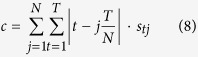


where 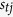 is the state of node *j* at time step *t*. Intuitively, it measures the “distance” to the diagonal and thus, the *lower* the value of *c* the more the process resembles a cascade.

Note that a given Boolean process can be made to look non-cascade like by shuffling the columns (the indices of the nodes). Thus, to compute the cascadeness distance, we first need a canonical transformation (permutation of columns) to enable meaningful comparisons. We do this through a smart-exhaustive algorithm that rearranges the columns to make a process as cascade-like as possible. The idea behind this algorithm is to swap columns heuristically to decrease the cascadeness distance, and to repeatedly do so until all possible combinations are enumerated. The detailed description of the algorithm is included in Supplementary information.

## Additional Information

**How to cite this article**: Chen, H. *et al.* Boolean Models of Biological Processes Explain Cascade-Like Behavior. *Sci. Rep.* **6**, 20067; doi: 10.1038/srep20067 (2016).

## Supplementary Material

Supplementary Information
