# Erratum: Boolean Models of Biological Processes Explain Cascade-Like Behavior

**DOI:** 10.1038/srep46387

**Published:** 2017-06-30

**Authors:** Hao Chen, Guanyu Wang, Rahul Simha, Chenghang Du, Chen Zeng

Scientific Reports 6: Article number: 20067; 10.1038/srep20067 published online: 01 29 2016; updated: 06 30 2017.

The original HTML version of this Article listed an incorrect volume number. This has now been corrected in the HTML version; the PDF version was correct at the time of publication.

