# Supplementary material for: Boolean Models of Biological Processes Explain Cascade-Like Behavior
Source: Sci Rep. 2016 Jan 29;7:20067. doi: 10.1038/srep20067 (PMC4731822; doi:10.1038/srep20067)
Supplement: Supplementary Information [file srep20067-s1.pdf]

Boolean Models of Biological Processes Explain Cascade-Like Behavior

Hao Chen[1,2], Guanyu Wang[1,3], Rahul Simha[4], Chenghang Du[1], Chen Zeng[1,5,*]
1 Department of Physics, The George Washington University, Washington, DC 20052, USA
2 Department of Chemistry, Wuhan Polytechnic University, Wuhan 430023, China
3 Department of Biology, South University of Science and Technology of China, Shenzhen 518055, China
4 Department of Computer Sciences, The George Washington University, Washington, DC 20052, USA
5 Department of Physics, Huazhong University of Science and Technology, Wuhan 430074, China
∗ Corresponding Author: Chen Zeng, E-mail: chenz@gwu.edu

Supplementary information

Boolean model

In Boolean network models, systems are discretized and $s_{ti} \in \{0,1\}$ represents the status of node i at time t. Then, in the next time step $t+1$, $s_{t+1,i}$ is only determined by the status of the nodes in the present time step, as shown in the following:

$$s_{t+1,i} = F_i(s_{t,1}, s_{t,2}, ..., s_{t,n}) \tag{1}$$

This class of models were first proposed by Kauffman for gene regulatory networks.[1] In those models, there were no restrictions on the regulation function F_i, and the networks were therefore *random Boolean networks*. In later years, Kauffman and others introduced specific rules, such as canalyzing rule[2] and threshold rule.[3] Threshold rule is more widely used and can be described in the following:

$$s_{t+1,i} = \begin{cases} 0 & \sum a_{ji}s_{tj} < \theta_i \\ 1 & \sum a_{ji}s_{tj} > \theta_i \\ s_{ti} & \sum a_{ji}s_{tj} = \theta_i \end{cases} \tag{2}$$

Here the interaction strength (or weight) a_{ji} takes a positive (or negative) value if node j activates (or inhibits) node i, respectively. Similarly, if a_{ii} takes a positive (or negative) value that means self-activation (or self-degradation). θ_i is the threshold and indicates the minimum value of the sum required for the activation. In particular, if we set all $\theta_i = 0$ and determine a_{ji} by the following rule: $a_{ji} = 1$ for activators, $a_{ji} = -\infty$ for repressors (Note: "$-\infty$" implies that the inhibition is dominant and it's dominant inhibition rule. If taking $a_{ji} = -1$, it's majority rule) and $a_{ii} = -0.5$ for self-degradation, Eq. (2) is equivalent to the following Boolean equation:[4]

$$s_{t+1,i} = \left(\sum_{j\neq i} s_{tj} \cdot g_{ji} + s_{ti}\overline{r_{ii}}\right) \prod_{j\neq i} \overline{s_{tj} \cdot r_{ji}}. \tag{3}$$

This equation is the starting point of this paper.

Algorithm for calculating the designability D and the minimality m

According to the definition, "designability" is the number of solutions of Eq. (3). Considering a process with size $N \times T$, since each s_{ti} (for $t = 2,3,...,T$ and $i = 1,2,...,N$) has a Boolean equation, totally we have $N \times (T-1)$ Boolean equations and the number of Boolean variables is $2N^2$ (N^2 for g_{ji} and the other N^2 for r_{ji}). At first, it appears challenging to solve. But all these $N \times (T-1)$ equations can be divided into N groups and in group i there is included the equations of $s_{2,i}, s_{3,i}, ..., s_{T,i}$. Now the number of equations in each group is only $T-1$. As can be seen, *the variables in each group are independent of variables in other groups*. Consequently, the "designability" is equal to the product of the number of solutions of each equation group. That is, $D = \prod_i d_i$, here d_i is the number of solutions of the equation group i and is also termed as the individual designability for node i.

Next, we consider how to calculate the individual designability d_i. Unfortunately, we have no better way than exhaustive enumeration and suspect that this problem is NP-hard, as the related minimality problem we proved was NP-hard.[5] But for a system with $N = 11$, the number of all possible solutions in each group is only $2 \times 3^{10} = 118,098$ and it's reasonable to compute this through enumeration.

For the minimality m, we can also use such a "divide and conquer" strategy. After solutions of each group are obtained, the solutions with the smallest number of edges can be identified. Then the sum of the number of edges of those solutions is the minimality m. More details are described in.[4,5]

Algorithm for calculating the cascadeness distance c

When calculating the cascadeness distance c, we need to find a best cascade-like rearrangement of the process. For a process with N columns, if each column is different, the number of all possible arrangements is $N!$. Particularly, if $N = 11$, then the number is $11! = 39,916,800$, the number is big but the enumeration is somewhat feasible.

We use a smart-exhaustive algorithm for enumeration. First, let us go back to the definition of c: $c = \sum_{j=1}^{N}\sum_{t=1}^{T}\left|t - j\frac{T}{N}\right| \cdot s_{tj}$: if we define $c_i = \sum_{t=1}^{T}\left|t - j\frac{T}{N}\right| \cdot s_{tj}$, then $c = \sum_{j=1}^{N} c_i$. It means that c is equal to the sum of the distance of each column. Second, for an arrangement P, if we can find a better arrangement by swapping the ith column and the jth column, then in the best arrangement the ith column and the jth column can't both be the same position of the arrangement P. The reason is very simple: swapping the ith column and the jth column can always lead to a better arrangement. This idea is applied to the enumeration, which we call the smart-exhaustive algorithm.

Fission yeast cell-cycle

Fig. 1 represents a system of 9 molecules involved in regulating the fission yeast cell-cycle.[6] In this system, self-activation of the node "Cdc2/13" is assumed for the purpose of simplification, otherwise, we need to add an all "1" node to active "Cdc2/13" constantly. Systems allowing for self-activations can be described in the following and there're only a small modification of the Eq. (3):

$$s_{t+1,i} = \left(\sum_{j\neq i} s_{tj} \cdot g_{ji} + s_{ti}\overline{r_{ii}} + \overline{s_{ti}}g_{ii}\right)\prod_{j\neq i}\overline{s_{tj} \cdot r_{ji}}. \tag{4}$$

Part A of the figure shows the Boolean process — the sequence of states during the cell-cycle. At the start, only molecules SK, Wee1, Ste9, and Rum1 are active; at each time step the status of individual molecules change and finally, after 9 steps the system settles into a steady state. Part B of the figure shows the underlying network that determines the Boolean process. As it turns out, this is not the only network that explains the process – the network shown in part C of the figure is a minimal (fewest possible edges) network that suffices. Moreover, if only keeping the stimulation edges of part C (as shown in part D), it clearly shows that the nodes are sequentially activated.

We asked whether or not the fission yeast cell-cycle process has a large designability as compared with other feasible processes with $N = 9$ and $T = 10$. The result is shown in Fig. 2a with the designability plotted against activity, where the size of each dot corresponds to the number of processes for that combination of activity and designability. One sees that the fission yeast cell-cycle process (the red triangle) has the largest designability among all the processes with $a = 0.32$ that we generated.

We also analyzed designability at the level of the individual molecules in the system. Because $D = \prod_i d_i$, it is possible that D is large but d_j is very small for some j. Then, small d_j would be a bottleneck that limits mutation stability. To reflect the true designability, we produce Fig. 2b, which is the same as Fig. 2a except that the x-axis now represents $d_{\min}$ (the smallest d_i, for $i = 1,2,\cdots,N$). One sees that the fission yeast cell-cycle process has higher $d_{\min}$ than almost all the sampled processes with $a = 0.32$.

Beyond the designability D, it is of interest to ask whether a process can be realized with an efficient network, that is, a network with few edges. In comparing two possible networks for the same process, one might reason that the network with more edges is harder for evolution to design. To see whether the fission yeast cell-cycle process is efficient in this sense, we compared it with one million randomly generated feasible processes of the same size ($N = 9$ and $T = 10$). For every process, we computed the number of edges in the minimal network needed for that process (the minimality m). As Fig. 3 shows, the fission yeast cell-cycle process (the red triangle) has much lower minimality (with $m = 18$) among all sampled processes, indicating that biological processes can be high efficiency designed.

References

1. Kauffman, S. A. Metabolic stability and epigenesis in randomly constructed genetic nets. *Journal of theoretical biology* **22**, 437–467 (1969).
2. Kauffman, S., Peterson, C., Samuelsson, B. & Troein, C. Random boolean network models and the yeast transcriptional network. *Proceedings of the National Academy of Sciences* **100**, 14796–14799 (2003).
3. Rohlf, T. & Bornholdt, S. Criticality in random threshold networks: annealed approximation and beyond. *Physica A: Statistical Mechanics and its Applications* **310**, 245–259 (2002).
4. Wang, G. *et al.* Process-based network decomposition reveals backbone motif structure. *Proc Natl Acad Sci USA* **107**, 10478–10483 (2010).

5. Wang, G. *et al.* Process-driven inference of biological network structure: Feasibility, minimality, and multiplicity. *PLoS ONE* **7**, e40330 (2012).

6. Davidich, M. I. & Bornholdt, S. Boolean network model predicts cell cycle sequence of fission yeast. *PLoS ONE* **3**, e1672 (2008).

Figure Legends

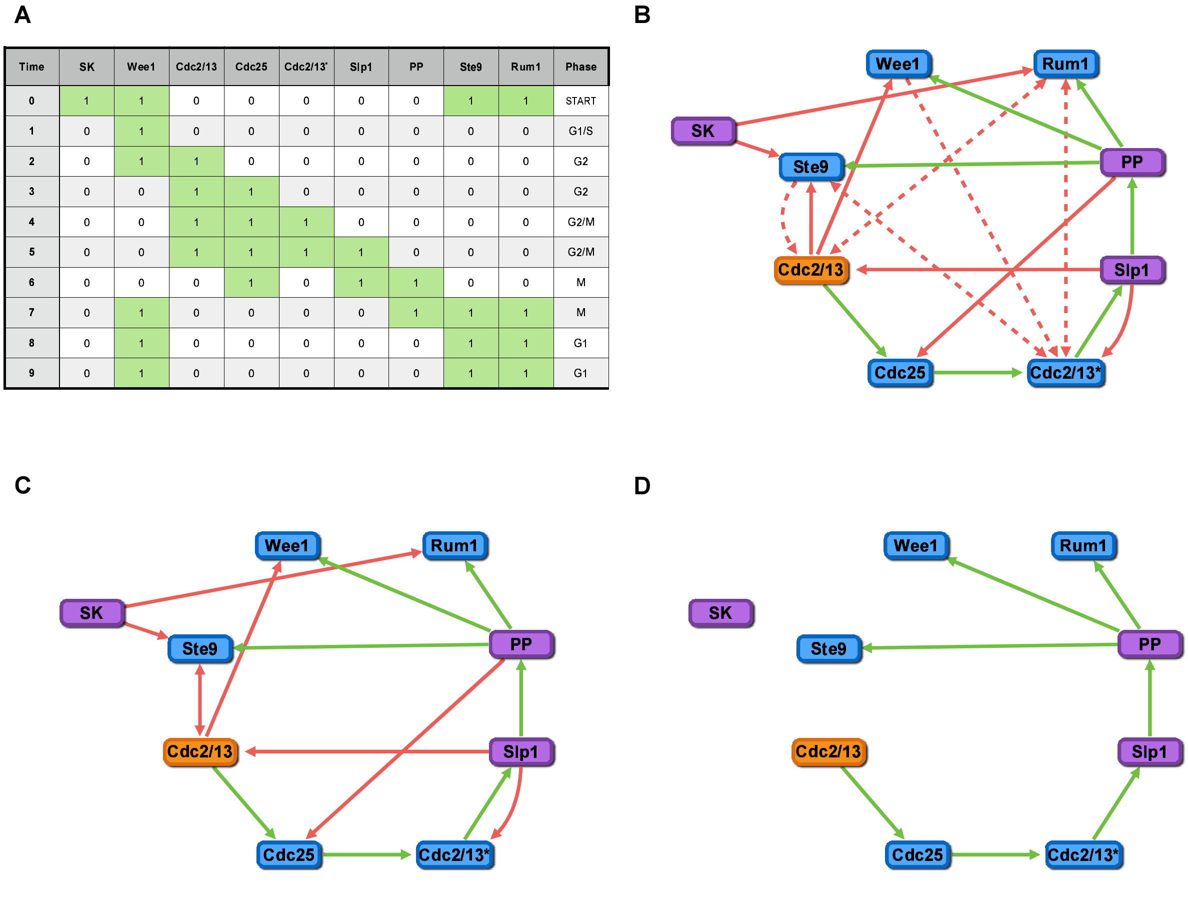

A

Time	SK	Wee1	Cdc2/13	Cdc25	Cdc2/13*	Slp1	PP	Ste9	Rum1	Phase
0	1	1	0	0	0	0	0	1	1	START
1	0	1	0	0	0	0	0	0	0	G1/S
2	0	1	1	0	0	0	0	0	0	G2
3	0	0	1	1	0	0	0	0	0	G2
4	0	0	1	1	1	0	0	0	0	G2/M
5	0	0	1	1	1	1	0	0	0	G2/M
6	0	0	0	1	0	1	1	0	0	M
7	0	1	0	0	0	0	1	1	1	M
8	0	1	0	0	0	0	0	1	1	G1
9	0	1	0	0	0	0	0	1	1	G1

Figure 1. A simplified description of the fission yeast cell-cycle. These figures are similar with the figures of the budding yeast cell-cycle in the text (Fig. (1)). And one difference is: self-activation is needed when modeling the cell-cycle of the fission yeast and we color that node ("Cdc2/13") to yellow in B-D. (A) The time course of the 9 nodes as a representation of the cell-cycle process. (B) The full cell-cycle network. (C) The backbone sub-network contained in the full network. (D) Stimulation edges only of the backbone sub-network.

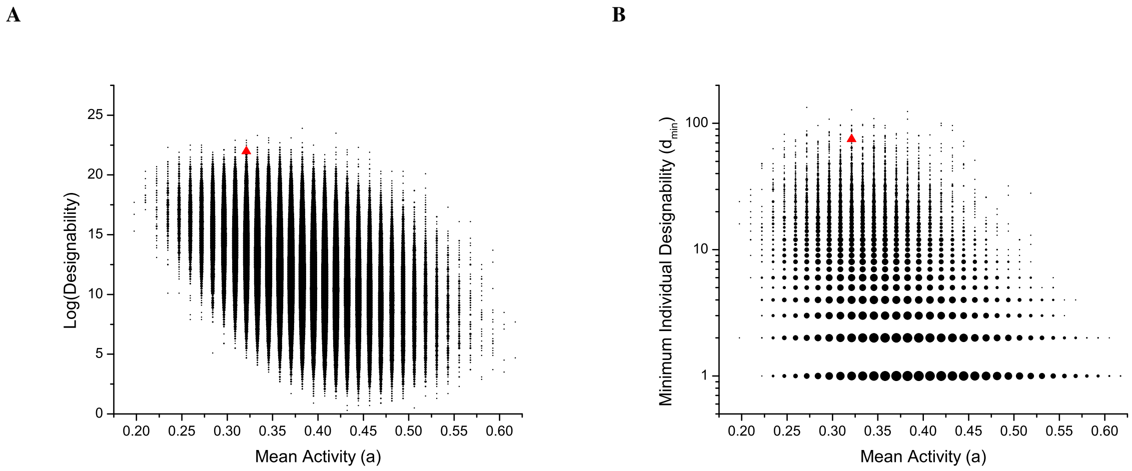

Figure 2. Designability versus mean activity of Boolean processes with $N = 9$ and $T = 10$. The size of each dot corresponds to the number of processes for that combination of mean activity and designability. The red triangle represents the fission yeast cell-cycle process. (A) The designability D in terms of Eq. (4). (B) The minimal individual designability d_{min} in terms of Eq. (4).

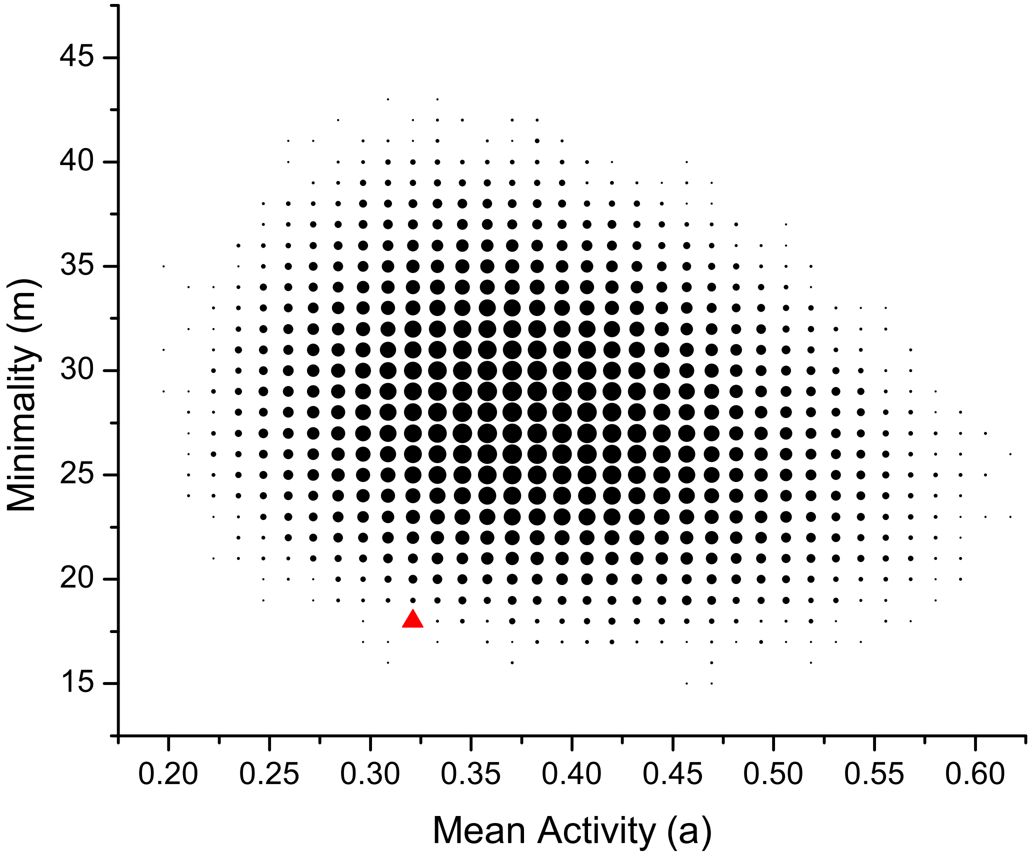

Figure 3. Minimality versus mean activity of Boolean processes with $N = 9$ and $T = 10$. The size of each dot corresponds to the number of processes for that combination of mean activity and minimality. Minimality is calculated under the Eq. (4). The red triangle represents the fission yeast cell-cycle process.

A B

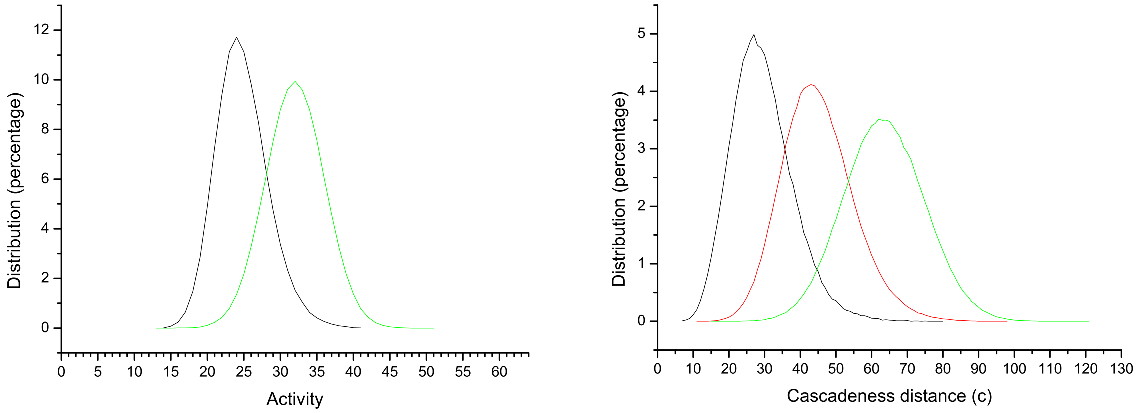

Figure 4. Distributions of the activity *A* and the cascadeness distance *c* of the selected 89,733 Boolean processes. We enumerate all Boolean processes of size 8x9 and all 112,877,531 robust processes are idenfied. Then the top 1% processes with regard to minimality *m*, designability *D* and minimum individual designability d_{min} are selected as the candidates for the biological sysems. The distribution of the activity *A* is shown in (A) with the black color and the random distribution is shown with the green color, it indicates that the selected processes have lower activity. Then we draw the figure of the distribution of the cascadeness distance *c* in (B). The black curve represents the selected processes, the red curve represents the random processes but with the same activity *A* distribution of the selected processes, and the green curve represents the totally random processes. It clearly indicated that the selected processes are much more cascade-like.
